# Photothermal-Responsive Polyvinyl Alcohol/Gelatin/Graphene Oxide Hydrogels Loaded with Quercetin for NIR-Triggered Controlled Drug Delivery

**DOI:** 10.3390/gels12040327

**Published:** 2026-04-14

**Authors:** Alexa-Maria Croitoru, Tatiana Tozar, Mihai Boni, Angela Staicu, Roxana-Doina Trușcă, Bianca-Maria Tihăuan, Anton Ficai

**Affiliations:** 1 Department of Science and Engineering of Oxide Materials and Nanomaterials, Faculty of Chemical Engineering and Biotechnology, National University for Science and Technology Politehnica Bucharest, Gh. Polizu St. 1–7, 011061 Bucharest, Romania; truscaroxana@yahoo.com; 2 National Centre for Food Safety, National University for Science and Technology Politehnica Bucharest, Spl. Independentei 313, 060042 Bucharest, Romania; ciubuca.b@gmail.com; 3 Laser Department, National Institute for Laser, Plasma, and Radiation Physics, 409 Atomistilor, 077125 Magurele, Romania; tatiana.alexandru@inflpr.ro (T.T.); mihai.boni@inflpr.ro (M.B.); angela.staicu@inflpr.ro (A.S.); 4 Extreme Light Infrastructure—Nuclear Physics, “Horia Hulubei” National Institute for R&D in Physics and Nuclear Engineering, 30 Reactorului Street, 077125 Magurele, Romania; 5 Research Institute of the University of Bucharest (ICUB), University of Bucharest, Spl. Independentei 91–95, 050095 Bucharest, Romania; 6 National Centre for Micro- and Nanomaterials, National University for Science and Technology Politehnica Bucharest, Spl. Independentei 313, 060042 Bucharest, Romania; 7 Academy of Romanian Scientists, 3 Ilfov Street, 050045 Bucharest, Romania

**Keywords:** smart hydrogels, photothermal therapy, on-demand drug delivery, antimicrobials, graphene oxide

## Abstract

Photothermal therapy (PTT) has emerged as a promising medical strategy for controlled and targeted drug delivery, due to its ability to trigger rapid release while minimizing damage to surrounding environments. Among different near-infrared (NIR)-responsive nanomaterials, carbon materials are of particular interest due to their multifunctional properties, with graphene oxide (GO) being a powerful photothermal therapy agent that can accelerate stimuli-responsive drug release. Herein, novel stimuli-responsive hydrogels based on polyvinyl alcohol (PVA), gelatin (Gel) and GO, loaded with natural quercetin (Q) were developed and evaluated for their physico-chemical properties, antibacterial and antifungal activities, photothermal Q release, and cellular metabolic activity. Upon NIR laser irradiation, after 10 min, Q was released twice as fast compared to conventional drug release without stimulation. The rapid release of Q by applying light radiation highlights the suitability of these hydrogels for controlled drug delivery applications. The PVA:Gel:GO/Q-hydrogels exhibited strong antimicrobial and antifungal performance (≥90% microbial reduction at higher GO concentrations). Furthermore, a significant reduction in *S. aureus* adhesion and invasion indicates the sample’s potential to mitigate bacterial infections. The PVA:Gel:GO/Q formulations exhibited high biocompatibility in Human Dermal Fibroblasts (HDF), demonstrating that Q improves the safety of PVA:Gel:GO-loaded hydrogels. These results offer promising potential for PVA:Gel:GO/Q hydrogels as advanced materials for photothermal-triggered drug delivery and antimicrobial applications.

## 1. Introduction

The biomedical sector has seen significant advancements with the development of stimuli-responsive materials capable of responding to external triggers such as light, heat, or pH, enabling controlled drug delivery and antimicrobial applications [1,2]. These materials have the ability to enable targeted, controlled delivery of active compounds at specific times and sites of interest [3,4,5,6]. In present-day medicine, designing new on-demand stimuli-responsive systems is essential in order to meet the need for safe and effective biomedical applications. Smart materials possess tailored responsive properties compared to conventional materials, thus enabling a wide range of applications, including drug delivery, tissue engineering, neurological disorders, etc. [7,8,9,10]. Over the last few years, photothermal therapy (PTT) has become a topic of major interest as a promising medical tool for controlled drug release and targeted antimicrobial applications [11,12,13]. PTT is a minimally invasive therapeutic technique that utilizes photothermal agents (PTAs)—such as nanoparticles, dyes, or other light-absorbing materials—to convert absorbed electromagnetic radiation, typically near-infrared (NIR) light, into localized heat. PTT is considered suitable for applications in the biomedical and pharmaceutical fields because it causes minimal heating damage to normal tissues, is noninvasive and target-specific, has antibacterial effects, and on-demand releases PTAs to the site of interest [14,15,16].

Lately, a variety of NIR-responsive dressings, such as sponges [17,18], microneedles [19,20], films [21], nanofibers [22,23], etc., have been developed, showing unique properties such as enabling deep tissue penetration with minimal harm to surrounding tissue. Among these dressings, hydrogel-based systems are considered a new promising class of PTAs due to their versatility and potential in advanced therapeutic platforms [24,25,26]. Hydrogels have a three-dimensional (3D) network structure that exhibits unique features, including hydrophilicity, biodegradability, biocompatibility, negligible cytotoxicity, antimicrobial protection, drug loading capacity, and tunable mechanical properties [27,28,29,30].

Because hydrogels have low mechanical strength, their functionalization with different nanomaterials can enhance the functional properties of the materials, including thermal, chemical, electrical, antibacterial, etc. [31]. Furthermore, incorporating different active ingredients (natural and/or synthetic) into their structure can enhance therapeutic efficacy and achieve improved treatment outcomes [32,33,34].

Among the various NIR-responsive nanomaterials, graphene oxide (GO) has shown great potential in biomedical applications due to its specific characteristics and its use as a powerful photothermal therapy agent. GO can absorb NIR radiation and rapidly release surface-bounded therapeutic agents upon NIR irradiation [35,36,37]. Moreover, as demonstrated in previous studies, the incorporation of GO in various hydrogel systems enhanced the mechanical properties by reinforcing the polymeric network through strong intermolecular interactions [21,38,39]. Photothermal-responsive GO-based hydrogels have the potential to eliminate cancerous cells, demonstrate excellent antibacterial effects, enable controllable drug release, and promote tissue repair [40,41,42]. For example, Lin et al. [43] investigated the antibacterial activity and controlled delivery of ibuprofen (IBU) from GO/sodium alginate (SA) hydrogel carrier under NIR light irradiation. The results revealed that the addition of GO into the hydrogel matrix increased the release rate of IBU under light irradiation, reaching 70% in 80 h. Moreover, the GO/SA/IBU hydrogel exhibited good antibacterial activity and excellent cytocompatibility. In the biomedical sector, there are different biodegradable polymers (collagen, poly(caprolactone), poly(lactic acid), chitosan, etc.) that have been used for biomedical applications. Among them, of special interest is polyvinyl alcohol (PVA), due to its particular properties. PVA is a biocompatible and non-toxic polymer with excellent properties, including chemical and mechanical strength, high hydrophilicity, and biodegradability [44,45,46]. Furthermore, gelatin (Gel)—a natural polymer derived from collagen by controlled hydrolysis—is suitable as a hydrogel platform because of its biodegradability, biocompatibility, exudate absorption, abundance, and low cost [47,48,49]. In the last few years, quercetin (3,3,4,7-pentahidroxiflavona), a natural bioflavonoid, has gained major interest in biomedical applications, owing to its antioxidant, anti-inflammatory, antimicrobial, and antitumoral properties [50,51]. Notwithstanding, quercetin (Q) has limited clinical applications because of its low bioavailability, poor stability, and sensitivity to light and heat. Thus, to overcome these problems, Q needs to be further functionalized [52].

**Figure 1:**
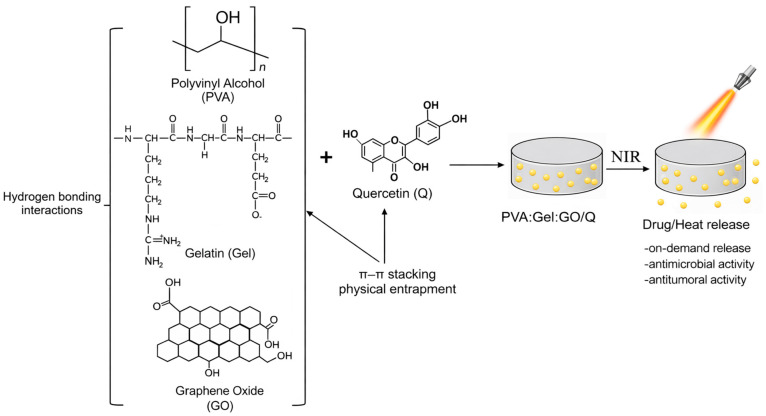
Schematic illustration of the experimental concept.

The scope of the current article is to develop and characterize novel stimuli-responsive PVA:Gel:GO/Q thermosensitive hydrogels for controlled drug delivery. In this regard, on-demand Q release from PVA:Gel:GO hydrogels is achieved, using NIR laser irradiation, which has the role of converting the light energy into local heat, releasing the therapeutics in a controlled way in the area of interest. By changing the concentration of photothermal agents, laser intensity, and irradiation time, the target temperature of the hydrogels can be adjusted [53,54]. The synthesized hydrogels were characterized to evaluate their physico-chemical properties, antibacterial activity, in vitro cytotoxicity, as well as the release profile of Q using NIR-laser irradiation. Figure 1 shows a schematic representation of the hydrogel system and the NIR-triggered drug release mechanism.

## 2. Results and Discussion

### 2.1. PVA/Gel Hydrogel Formation and Structure

The formation of PVA:Gel hydrogels involves both physical and chemical crosslinking mechanisms. By crosslinking PVA:Gel hydrogels with GA, the physico-chemical properties such as membrane permeability, mechanical strength, stability in aqueous solutions, degradation rate, etc., can be enhanced, making the hydrogels more resistant to degradation while maintaining their structural integrity [55,56].

As reported in previous studies [57,58,59], GA is known for reacting mainly with the amino groups (-NH_2_), forming Schiff base linkages. Based on the results of the present study, PVA:Gel 30:70 hydrogel was selected, because the higher Gel concentration resulted in stronger gel formation and a more interconnected polymer network. While GA interacts more with Gel molecules, PVA mainly participates through physical interactions within the polymer matrix. PVA was incorporated to improve the overall performance of the Gel hydrogel. Based on previous studies reported in the literature, by optimizing the proportions of PVA:Gel together with the crosslinking agent, advanced biomaterials suitable for biomedical applications can be developed [60,61,62].

During the freeze–thaw process of the hydrogels, the physical crosslinking of PVA and Gel molecules occurred, forming a combined polymer network with reinforced physical properties [63]. Furthermore, the stability of the hydrogel matrix increased, due to additional hydrogen bonding interactions between PVA hydroxyl groups and Gel functional groups [64,65].

**Figure 2:**
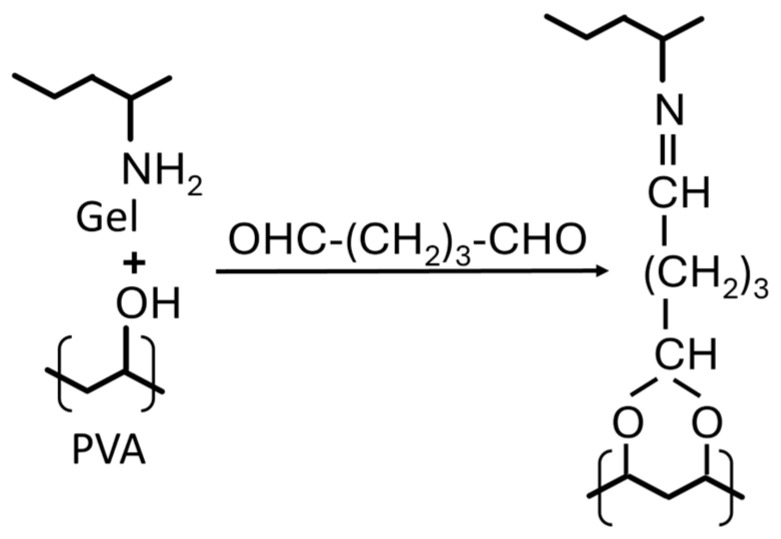
Chemical crosslink reactions of PVA:Gel crosslinked with GA.

Due to the complexity of the reaction mechanism between GA and protein, according to Matsuda et al. [66], there is a possibility that some aldehyde groups may remain unreacted. Thus, by adding GA in a PVA/Gel solution, a reaction between the hydroxyl functional groups of the PVA chains and the amino groups (-NH_2_) of the Gel can be possible, forming a crosslinked co-polymer. Figure 2 shows the chemical reactions of PVA/Gel crosslinked with GA.

### 2.2. Fourier Transform Infrared Spectroscopy—FTIR

**Figure 3:**
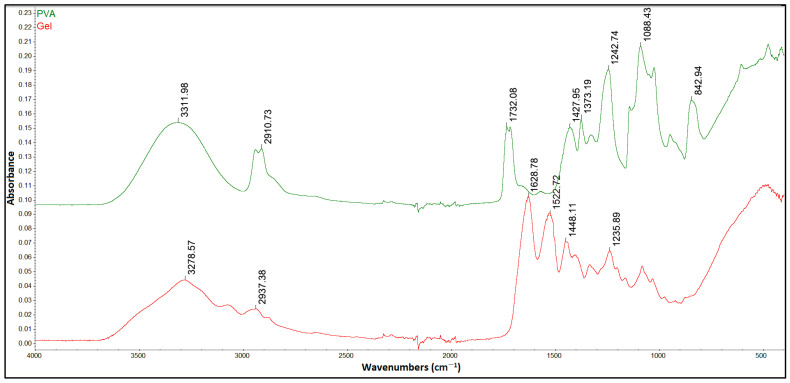
FTIR spectra of pristine PVA and Gel.

Figure 3 shows the characteristic peaks of PVA and Gel. The FTIR spectra of pure PVA show the absorption peak at 3311 cm^−1^ belonging to the O-H stretching vibration, and the peak at 2910 cm^−1^ characteristic of the asymmetric stretching of CH_2_. The peak at 1732 cm^−1^ indicates the presence of C=O due to the strong carboxylic group. The C-H bending vibration of CH_2_ groups occurs at 1427 cm^−1^, and the peak at 1373 cm^−1^ corresponds to C–H deformation vibration. The presence of the peaks at 1088 and 824 cm^−1^ is attributed to C–O stretching of acetyl groups and C-C stretching vibration, respectively [67,68].

The characteristic peaks of Gel polymer were observed at 3278 cm^−1^, representing the N-H stretching vibration of the amides II band, and at 2937 cm^−1^, showing the C-H stretching bond. The three characteristic signals at 1628, 1522, and 1235 cm^−1^ correspond to the C=O stretching vibration and amide I, N-H bending vibration, and amide III stretching vibration, respectively [69,70].

**Figure 4:**
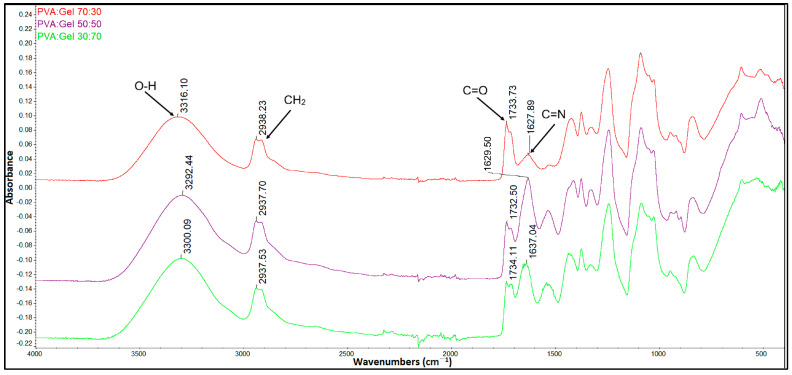
FTIR spectra of PVA/Gel hydrogels in different ratios.

Figure 4 shows the FTIR spectra of PVA:Gel hydrogels in different ratios. The broad peak that appears in the region 3000 and 3600 cm^−1^, characteristic of the O-H stretching vibration, indicates the chemical interaction between PVA and Gel. With a higher amount of Gel, the specific absorption band of PVA around 1730 cm^−1^ decreased in intensity. The bands between 1000 and 1100 cm^−1^ can be assigned to acetal formation (O–C–O vibration) resulting from the reaction between aldehyde groups of GA and hydroxyl groups of PVA. Moreover, the band around 1630 cm^−1^ indicates the formation of imine groups (C=N), which can be related to the interaction of the amine group of gelatin and aldehyde groups of GA. Because the FTIR spectrum lacks the characteristic peak of GA (C=O at 1700 cm^−1^), it can indicate that GA was neutralized during the preparation process [56,70,71].

**Figure 5:**
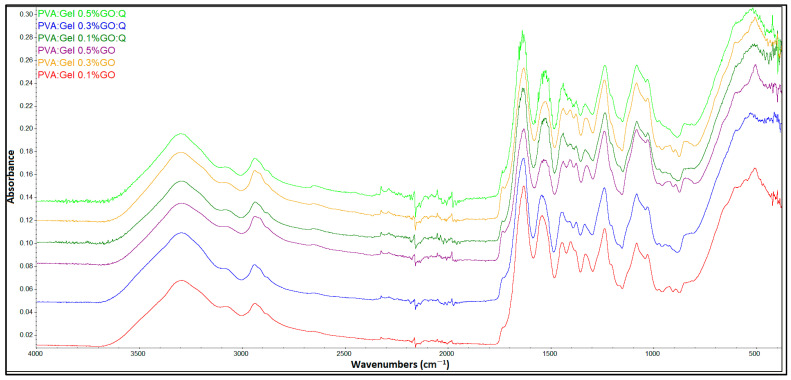
FTIR spectra of PVA:Gel 0.1%GO, PVA:Gel 0.3%GO, PVA:Gel 0.5%GO, and PVA:Gel:GO:Q hydrogels.

Figure 5 shows FTIR spectra of PVA:Gel 0.1%GO, PVA:Gel 0.3%GO, PVA:Gel 0.5%GO, and PVA:Gel:GO:Q hydrogels. FTIR spectra of GO and Q, showing the main characteristic peaks, are reported in our previous literature reports [50,72]. With the addition of GO, a decrease in the relative intensity of the peak around 1630 cm^−1^ can be observed, highlighting the interaction of GO with Gel. Moreover, the peak at 1637 cm^−1^ from PVA:Gel 30:70 shifted to 1629 cm^−1^, suggesting the bonding between GO and Gel [73]. After the loading of hydrogels with Q, no important changes were observed, with the hydrogels having an apparently irregular surface. Comparing Q-loaded hydrogels, a decrease in the relative peak intensity is observed with increasing GO concentration. Moreover, comparing Q-loaded hydrogels with PVA:Gel:GO hydrogels, an increase in the relative intensity of the bands can be seen due to the addition of Q into the hydrogel structure.

### 2.3. Morphological Examination

**Figure 6:**
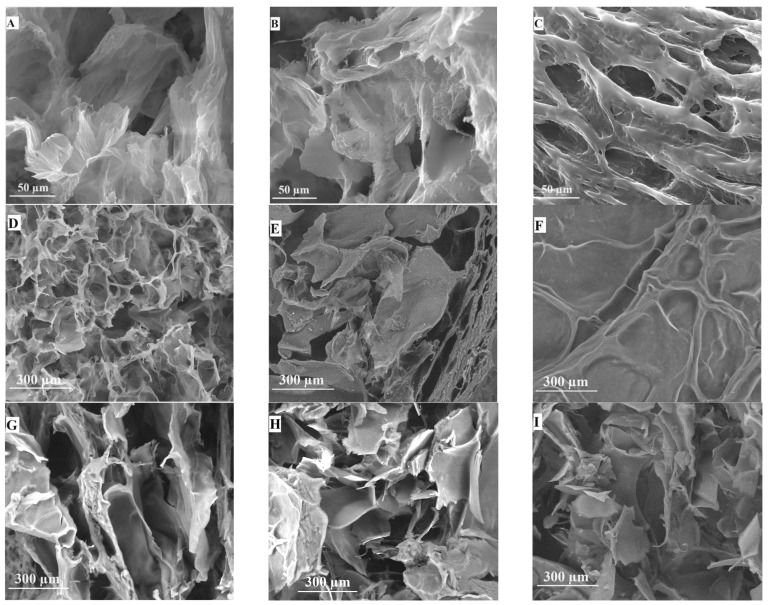
Surface morphology at 2000× magnification of: (**A**) PVA:Gel 30:70, (**B**) PVA:Gel 50:50, and (**C**) PVA:Gel 70:30; and at 500× magnification of: (**D**) PVA:Gel 0.1% GO, (**E**) PVA:Gel 0.3% GO, (**F**) PVA:Gel 0.5% GO, (**G**) PVA:Gel 0.1% GO/Q, (**H**) PVA:Gel 0.3% GO/Q, and (**I**) PVA:Gel 0.5% GO/Q.

The morphology of PVA:Gel hydrogels was studied using SEM microscopy. The results of microscopic measurements are shown in Figure 6. Although all the materials show an amorphous structure (revealing the formation of various pore geometries and dimensions along the cross-section area), some differences between the hydrogels can be observed (Figure 6A–C). The PVA:Gel 70:30 has the smallest pore size at the same magnification. An increase in the Gel content of the hydrogel blends led to larger pore sizes [70,71,74,75].

Pore size distribution was carried out for each material sample on 10 such images using ImageJ 1.54g software (NIH, Bethesda, MD, USA). The measurement indicated that hydrogels with higher PVA content had smaller pores. The average pore size for PVA:Gel 70:30 was 13.38 µm, for PVA:Gel 50:50 was 28.05 µm, and for PVA:Gel 30:70 was 36.03 µm [70].

Figure 6D–I also shows a porous structure with a sponge-like aspect and irregular pore size distribution. After the incorporation of GO, the pore diameter increased with the increase in the GO content and varied between 31.92 µm and 86.70 µm. The morphological differences can be observed in PVA:Gel 0.5% GO hydrogel due to the increased dispersion of GO within the polymer matrix, establishing weak intermolecular interactions [76,77,78]. After loading the Q, the average pore diameter was approximately 45 µm across all hydrogels. A high concentration of Q in the polymer matrix appears to lead to an uneven sponge-like structure of the hydrogels. The obtained results show evident crosslinking between GO material and polymeric matrix, revealing a porous structure suitable for releasing the active compound Q [79,80].

### 2.4. Water Content in Hydrogels

Table 1 presents the content of the water in PVA:Gel hydrogels. The results indicate that the water content of PVA:Gel hydrogels increased with increasing Gel content. This can be related to the hydrophilic functional groups of PVA and Gel, which can easily form hydrogen bonds with water molecules [81]. Compared to PVA:Gel 30:70, the water content in PVA:Gel:GO and PVA:Gel:GO/Q hydrogels slightly decreased from 96.72% to approximately 90% in all samples. Overall, the obtained hydrogels have a good water retention, ranging from 88.66% to 96.72% [82,83].

### 2.5. Swelling Behavior of PVA:Gel:GO Hydrogels

**Figure 7:**
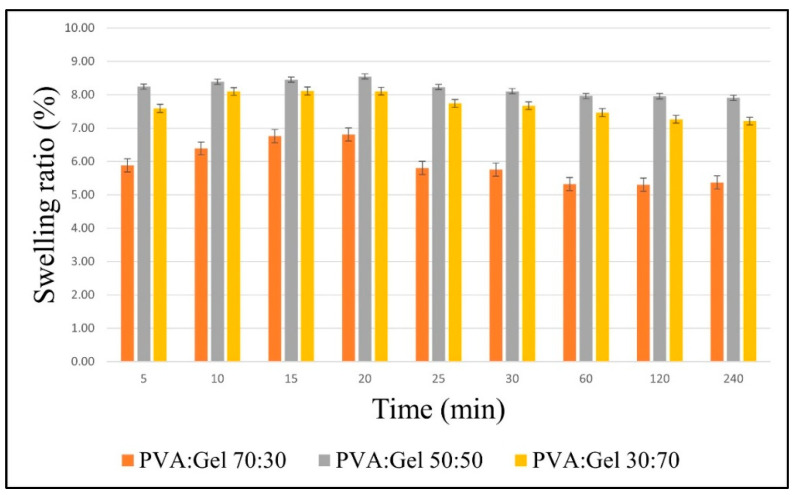
Swelling ratio of PVA:Gel at different concentrations in SWF at 37 °C.

Swelling behavior was evaluated to obtain a good drug delivery system and to investigate the stability of hydrogels. This characteristic indicates the capabilities of absorbing fluids and exudates. Figure 7 shows the swelling behavior of PVA:Gel hydrogels, measured at 37 °C in simulated wound fluid (SWF). According to literature data, PVA:Gel hydrogels have good fluid absorption capacity, because of their hydrophilic functional groups: hydroxyl (–OH), amino (–NH_2_), and carboxyl (–COOH) with very significant hydrogen bonding [63,84]. The prepared hydrogels exhibited rapid swelling, reaching equilibrium within 30 min. The PVA:Gel 70:30 hydrogels have the least fluid absorption, with a maximum value of 6.81% at 20 min. When the Gel concentration was increased gradually, the fluid uptake increased, due to the Gel’s higher swelling capacity, because of the porosity in its network structure. The maximum value for PVA:Gel 50:50 was reached at 20 min (8.55%), and for PVA:Gel 30:70 was 8.11% at 15 min. After 60 min, the degradation behavior started, in particular for PVA:Gel 70:30 and PVA:Gel 50:50, which were completely degraded after 24 h. The weight loss for the PVA:Gel 30:70 hydrogel decreased after day 5. This can be due to the increase in the size of the pores and to the hydrogel structure that begins to open, resulting in a reduction in the rigidity of the Gel [70,84].

**Figure 8:**
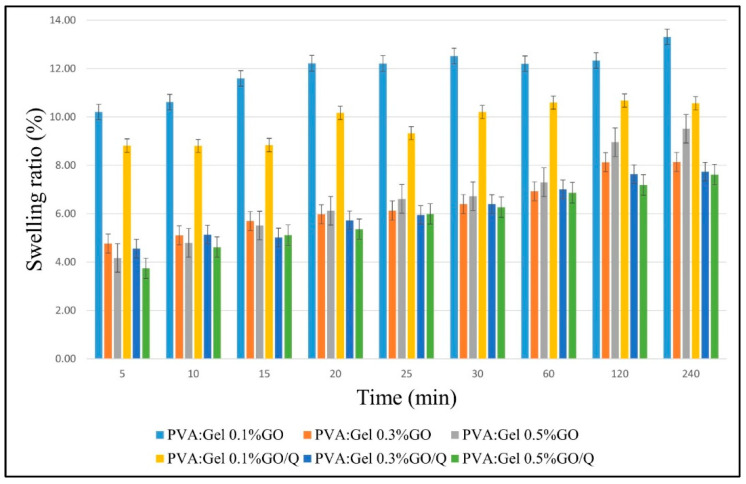
Swelling ratio of PVA:Gel/GO and PVA/Gel/GO/Q hydrogels in SWF at 37 °C.

Figure 8 illustrates the fluid uptake of PVA:Gel:GO and PVA:Gel:GO/Q hydrogels. After immersion of the hydrogels in SWF, the swelling ratio was determined at various time intervals. Compared to the swelling behavior of PVA:Gel 30:70 (Figure 7), the addition of GO, containing a large number of functional groups, results in an increased number of polar groups and, thus, an increase in fluid uptake. Moreover, with the incorporation of Q into the formulations, a slight decrease in the swelling rate can be observed due to the hydrophobic nature of the Q. Nonetheless, the fabrication of these hydrogels has enhanced solubility and stability while reducing degradation. Thus, the samples loaded with Q demonstrated good swelling capacity, with maximum values of 10.68% for PVA:Gel 0.1%GO/Q, 7.74% for PVA:Gel 0.3%GO/Q, and 7.62% for PVA:Gel 0.5%GO/Q in 4 h. The PVA:Gel 0.1%GO, PVA:Gel 0.3%GO, and PVA:Gel 0.5%GO were completely degraded on day 5. The weight loss for the hydrogels with Q started after day 9 [85,86]. The data are presented as mean ± standard deviation (SD) of three independent experiments.

### 2.6. In Vitro Drug Release

**Figure 9:**
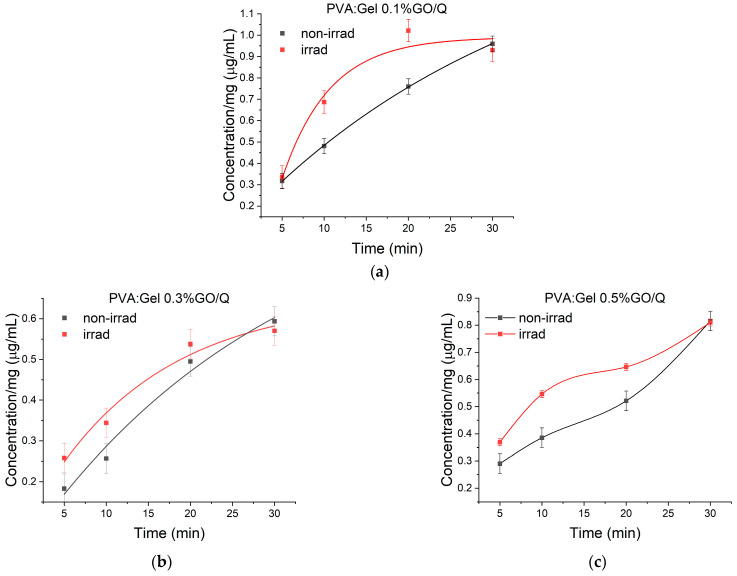
Time evolution of the amount of Q released per mg of hydrogel in PBS by non-irradiated and irradiated hydrogels with different GO concentrations: (**a**) 0.1%, (**b**) 0.3%, and (**c**) 0.5%; the error bars represent the standard error of the mean.

A 785 nm laser diode was used to study the photothermal effects of the hydrogels for exposure intervals of 5, 10, 20, and 30 min. Figure 9 presents the cumulative release of Q from the PVA:Gel:GO/Q hydrogels in phosphate-buffered saline (PBS) solution with and without laser irradiation. Upon laser irradiation, GO nanoparticles convert light into heat, potentially triggering the localized release of Q from the GO surface.

For PVA:Gel 0.1%GO/Q, the release rate of Q was approximately the same in the first 5 min of incubation [63,87]. With the increase in the GO concentration, an accelerated release of the drug was achieved when laser stimulation was applied at 0.26 µg/mL for the hydrogel with 0.3%GO and 0.37 µg/mL for the hydrogel with 0.5%GO compared to 0.18 µg/mL for PVA:Gel 0.3%GO/Q and 0.29 for PVA:Gel 0.5%GO/Q without any stimulation. The lowest proportion of Q release was observed for PVA:Gel 0.3%GO/Q, with a gradual increase up to 30 min. The highest difference in release capacity was recorded after 10 min of irradiation, with a release rate approximately twice as fast as the conventional drug-release method in the absence of stimulation, indicating that laser irradiation accelerates the initial release of Q. After 30 min, the released concentrations became similar, suggesting that both systems approached a release plateau. Therefore, the main role of laser irradiation, in our study, was to enhance the early release kinetics rather than the final amount released. Furthermore, the irradiation power density used in this study (0.0298 W cm^−2^) is significantly lower than that typically used in photothermal therapy (>0.5 W cm^−2^). Sun Z et al. [88] reported that the temperature of the hydrogel increased to 79.5 °C at 1.0 W cm^−2^ and to 88.2 °C at 1.5 W cm^−2^. Accordingly, only a modest temperature increase is expected under our experimental conditions. In future studies, we will aim to reach temperatures suitable for photothermal therapy and perform direct infrared thermography measurements to quantify hydrogel temperature changes during irradiation. The rapid release of Q at the target site, by applying light radiation, may provide improved drug efficiency and therapeutic effectiveness. The obtained results demonstrated the photothermal efficiency of GO in hydrogels. This is an important feature of GO as a PTA for applications toward light-induced PTT [89].

### 2.7. Antimicrobial Activity of PVA:Gel:GO Hydrogels

The tested samples were developed by incorporating GO and Q at different concentrations into a PVA:Gel matrix, a biomaterial previously reported as suitable for diverse biomedical applications due to its hydrophilicity, elasticity, stability, and capacity to enhance the antimicrobial activity of loaded agents [90]. In our previous work [91], the GO/Q combination demonstrated potent antimicrobial effects, achieving microbial reduction rates exceeding 90%.

**Figure 10:**
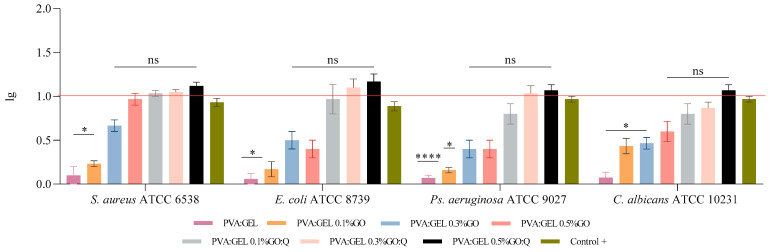
Logarithmic reduction of *S. aureus* ATCC 6538, *P. aeruginosa* ATCC 15442, *E. coli* ATCC 8739, and *C. albicans* ATCC 10231 strains by tested samples; Control + represents Clindamycin for *S. aureus*, Trimethoprim-sulfamethoxazole for *E. coli*, Gentamicin for *Ps. Aeruginosa*, and Fluconazole for *C. albicans*; * *p* value <0.05 (*n* = 3), **** *p* value <0.0001 (*n* = 3); ns = non-significant statistically.

In this study, the antibacterial efficacy of the samples was assessed against clinically relevant pathogens: *S. aureus* ATCC 6538, *Pseudomonas aeruginosa* (*P. aeruginosa*) ATCC 15442, *Escherichia coli* (*E. coli*) ATCC 8739, and *Candida albicans* (*C. albicans*) ATCC 10231. For each strain, antibiotic controls were included—clindamycin for *S. aureus*, trimethoprim-sulfamethoxazole for *E. coli*, gentamicin for *P. aeruginosa*, and fluconazole for *C. albicans*—and considered the benchmark for effective microbial reduction. The benchmark is represented in Figure 10 by a horizontal red line indicating the activity of the antibiotic control.

The obtained results highlight strong antimicrobial and antifungal performance, with several formulations surpassing the 1-log reduction threshold, corresponding to ≥90% decrease in microbial populations (Figure 10). Among bacterial strains, *S. aureus* exhibited the highest susceptibility, with log reductions of 0.96 ± 0.11 for PVA:Gel 0.5%GO, 1.03 ± 0.05 for PVA:Gel 0.1%GO/Q, 1.05 ± 0.06 for PVA:Gel 0.3%GO/Q, and 1.11 ± 0.07 for PVA:Gel 0.5%GO/Q. In the case of *E. coli*, the most efficient formulations were PVA:Gel 0.3%GO/Q (1.10 ± 0.17) and PVA:Gel 0.5%GO/Q (1.16 ± 0.15). Against *P. aeruginosa*, PVA:Gel 0.5%GO/Q demonstrated notable activity, reaching 1.06 ± 0.11 log reduction.

Regarding antifungal activity, PVA:Gel 0.5%GO/Q achieved the most pronounced effect (1.05 ± 0.11), though PVA:Gel 0.1%GO/Q (0.80 ± 0.20) and PVA:Gel 0.3%GO/Q (1.03 ± 0.15) also displayed meaningful inhibitory potential. These findings suggest that the embedding of GO and Q into the PVA:Gel matrix provides a synergistic antimicrobial benefit, particularly at higher concentrations, with broad activity across both bacterial and fungal strains. All antimicrobial experiments were performed on non-irradiated hydrogels to assess their intrinsic antimicrobial activity independent of photothermal stimulation.

When compared with previous reports, our findings reinforce the growing evidence that GO, particularly when combined with quercetin, exerts synergistic antimicrobial effects by disrupting microbial membranes and interfering with intracellular processes [92,93]. Notably, the efficacy observed across both Gram-positive and Gram-negative bacteria, as well as fungi, indicates broad-spectrum applicability, consistent with recent studies investigating nanocomposite-based antimicrobial systems [91,94].

Overall ranking of antimicrobial efficacy is PVA:Gel 0.5%GO/Q > PVA:Gel 0.3%GO/Q > PVA:Gel 0.1%GO/Q > PVA:Gel 0.5%GO. These ranking highlights that formulations combining both GO and Q were consistently more effective than GO alone, with PVA:Gel 0.5%GO/Q achieving the most pronounced reductions across all tested strains. PVA:Gel 0.3%GO/Q also demonstrated robust activity, particularly against *E. coli* and *C. albicans*. PVA:Gel 0.1%GO/Q showed moderate but noteworthy antimicrobial performance, while PVA:Gel 0.5%GO, although effective against *S. aureus*, was comparatively less potent than the GO/Q composites.

Overall, the combination of GO and Q, particularly at higher concentrations, appears to provide superior antimicrobial efficacy compared to GO alone, suggesting a synergistic interaction that enhances the therapeutic potential of the PVA:Gel matrix.

### 2.8. Bacterial Association and Invasion on Fibroblasts

**Figure 11:**
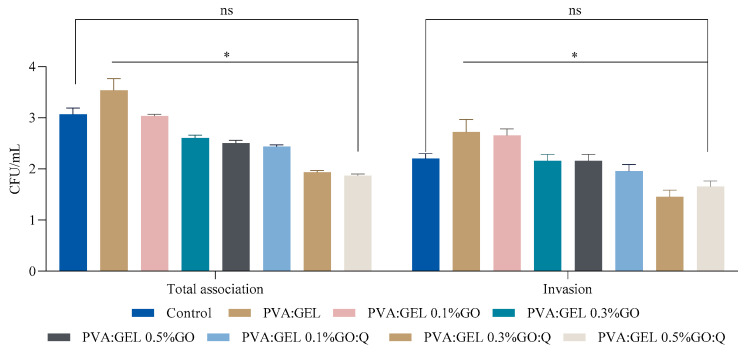
Effect of samples on total association and invasion of *S. aureus* ATCC 6538 strains in fibroblasts. * *p* value < 0.05 (*n* = 3); ns = non-significant statistically.

A significant reduction in the total association and invasion of *S. aureus* was observed in the presence of the samples tested, consistent with their demonstrated antimicrobial activity (Figure 11). This decrease in bacterial association and invasion may be attributed to the samples’ ability to inhibit bacterial adhesion, disrupt biofilm formation, and impair the mechanisms of bacterial invasion into host cells. These findings indicate that the samples may exert their effects either through direct bactericidal or bacteriostatic activity or by interfering with key virulence factors essential for host–pathogen interactions. The observed correlation between decreased association/invasion and antimicrobial efficacy highlights the sample’s potential as an effective agent for mitigating *S. aureus* infections through inhibition of bacterial adherence and invasion.

### 2.9. In Vitro Cytotoxicity of PVA:Gel:GO Hydrogels

In our previous study [91], GO and GO/Q composites demonstrated biocompatibility at lower concentrations; however, higher concentrations exhibited cytotoxic effects. For instance, GO/Q at 2.5% induced cytotoxicity in both normal fibroblasts (L929) and breast cancer cells (BT474), with a more pronounced effect on fibroblasts. The therapeutic index for GO/Q 2.5% was calculated as 1.417, indicating a narrow window between effective and toxic doses, highlighting the critical importance of precise dosing to avoid adverse effects. Building on these findings, we optimized the formulations by lowering GO and Q concentrations, slowing and controlling release kinetics, and enhancing overall biological functionality.

**Figure 12:**
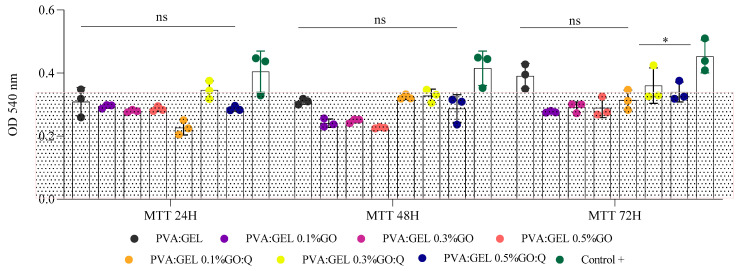
Biocompatibility assessment on HDF cells by MTT on PVA:Gel:GO/Q samples at 24, 48, and 72 h of contact; C+ = control − untreated cell model for normal response; * *p* value < 0.05; ns = statistically non-significant. The dotted area indicates the range of cell viability considered acceptable for biocompatibility.

Q appears to mitigate the cytotoxicity associated with GO, particularly at 0.3% concentration, as reflected by higher MTT values compared to GO alone. In contrast, higher GO concentrations (0.5%) showed slightly reduced cell viability, indicating a dose-dependent cytotoxic effect (Figure 12). Baseline PVA: Gel without GO exhibited only a minor reduction in cell viability (87.66 ± 16.16% at 72 h), confirming the inherent biocompatibility of the matrix. The addition of 0.1% GO to PVA: Gel resulted in a more pronounced decrease in viability (62.00 ± 7.55%), likely due to oxidative stress, membrane disruption, or ROS generation.

Notably, the incorporation of Q at 0.3% and 0.5% (PVA:Gel:GO/Q) improved cell viability to 79.33 ± 4.72% and 76.33 ± 14.29%, respectively, with the 0.3% GO/Q formulation exhibiting the highest viability at 72 h. This protective effect is likely attributable to the well-established antioxidant properties of Q, which can scavenge reactive oxygen species and reduce cellular damage.

**Figure 13:**
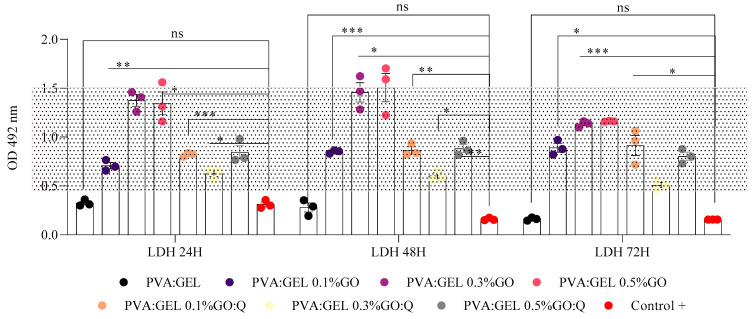
Cytotoxicity assessment by LDH on PVA:Gel:GO/Q samples at 24, 48, and 72 h of contact; C+ = control − untreated cells model for normal LDH release; * = *p* value < 0.05, ** *p* < 0.01, *** *p* < 0.001; ns = statistically non-significant. The dotted area represents the baseline LDH release corresponding to the control (untreated cells).

LDH assay results correlated with the MTT findings. All PVA:Gel:GO formulations showed elevated LDH release, indicative of cytotoxic stress, whereas PVA:Gel:GO/Q samples demonstrated reduced LDH release (Figure 13). Among these, the PVA:Gel 0.3%GO/Q formulation exhibited the lowest LDH release compared to the control, further supporting the role of quercetin in enhancing biocompatibility and mitigating the cytotoxic effects of GO.

The therapeutic index (TI) for each formulation was calculated as the ratio between the highest non-cytotoxic concentration, determined from MTT assays on Human Dermal Fibroblasts (cell viability ≥ 70%), and the minimum concentration required to achieve a ≥1-log reduction (≥90% reduction) in microbial populations for each tested strain. TI values thus reflect the relative safety margin between effective antimicrobial activity and cytotoxicity.

The TI values (Table 2) highlight the relative safety and efficacy of the GO and GO/Q-loaded PVA:Gel scaffolds. Across all tested strains, the addition of Q generally improved the TI compared to GO alone, indicating a more favorable balance between antimicrobial potency and cytocompatibility. For instance, the TI for *S. aureus* increased from 0.67 ± 0.60 for PVA:Gel:GO to 0.71 ± 0.58 for PVA:Gel:GO/Q, while *C. albicans* showed a similar trend (0.70 ± 0.62 → 0.79 ± 0.65). These enhancements are likely attributable to Q’s antioxidant and cytoprotective properties, which reduce GO-induced oxidative stress in host cells, without compromising antimicrobial activity.

Interestingly, the TI for *E. coli* decreased from 2.21 ± 1.97 in the GO-only scaffold to 0.95 ± 0.78 in the GO/Q formulation. This may reflect that the effective antimicrobial concentration of GO/Q against *E. coli* is higher relative to its cytotoxic threshold, emphasizing the strain-specific nature of the therapeutic window. Overall, the TI values indicate that while all scaffolds provide antimicrobial activity, GO/Q formulations, particularly at optimized concentrations, can enhance biocompatibility while maintaining efficacy, with PVA:Gel 0.3%GO/Q representing the most balanced profile across multiple microbial strains. Although hemocompatibility is an important parameter for translational applications, the present study focused on cytocompatibility with dermal fibroblasts, as hydrogels are intended for topical use. Future studies will include hemocompatibility evaluation and in vivo investigations to further assess clinical safety and validate the therapeutic potential of these hydrogels under physiological conditions.

## 3. Conclusions

In this paper, novel PVA:Gel:GO hydrogels loaded with natural Q were successfully designed. The physico-chemical properties, release profile, and antimicrobial activity were investigated. GO was synthesized by the modified Hummers method from graphite powder. Regarding the swelling behavior, the fluid uptake increased with the increase in Gel concentration, reaching a maximum value of 8.55% for PVA:Gel 50:50 after 20 min. For PVA:Gel:GO and PVA:Gel:GO/Q hydrogels, the addition of GO leads to an increased fluid uptake, demonstrating improved solubility and stability. Moreover, the hydrogels without Q were completely degraded after day 5, but the weight loss for the hydrogels with Q started after day 9.

GO with great photothermal efficiency can release Q in a controlled way using laser irradiation. When 785 nm laser stimulation was applied, a faster release of the drug can be seen with the enhancement of the GO concentration (0.26 µg/mL for PVA:Gel 0.3%GO/Q and 0.37 µg/mL for PVA:Gel 0.5%GO/Q). After 10 min of irradiation, the release capacity was approximately twice as fast as the conventional drug-release method in the absence of stimulation. These results showed that the hydrogels have the potential for noninvasive laser-triggered controlled drug release. It is worth mentioning that the PTT effect is suitable for modulating the Q release profile.

Incorporating GO and Q into a PVA:Gel matrix produces composites with potent antimicrobial and antifungal activities against pathogens of clinical relevance, including *S. aureus*, *E. coli*, *P. aeruginosa*, and *C. albicans*. Several formulations surpassed the 1-log reduction threshold, corresponding to ≥90% microbial reduction, with PVA:Gel 0.5% GO/Q consistently showing the strongest activity across all strains. The high cell survival observed in the baseline PVA:Gel formulation (87.66 ± 16.16%) confirms the intrinsic biocompatibility of the matrix. The addition of GO led to a dose-dependent decrease in viability, with 0.3% GO exhibiting significant cytotoxicity (62.00 ± 7.55%), likely due to oxidative stress and ROS generation. Incorporation of Q, particularly in the 0.3% GO/Q formulation, substantially improved cell survival (79.33 ± 4.72%), highlighting its ability to mitigate GO-induced cytotoxicity. This protective effect is likely attributable to Q’s antioxidant properties, which reduce reactive oxygen species production and protect cells from oxidative damage. LDH assay results corroborated these findings, showing decreased LDH release in PVA:Gel:GO/Q samples. Overall, the PVA:Gel 0.3% GO/Q formulation exhibited the highest biocompatibility, demonstrating that Q effectively enhances the safety profile of GO-loaded hydrogels. Future studies will include hemocompatibility evaluation and in vivo investigation to further assess clinical safety and validate the therapeutic potential of these hydrogels under physiological conditions.

Although GO-hydrogels exhibit good physico-chemical, photothermal, and antimicrobial properties, there are still some limitations in PTT, such as potential cytotoxicity, unintended thermal damage, insufficient therapeutic effect, etc. Therefore, there is a need to develop and improve novel materials that address their issues by increasing the biocompatibility of GO-hydrogels and improving targeting ability.

## 4. Materials and Methods

### 4.1. Materials

Glycine (CAS: 56-40-6) was purchased from Riedel del Haen, Hanover, Germany. PVA Mw ~130.000 (CAS: 9002-89-5), gelatin from porcine skin (Gel) (CAS: 9000-70-8), glutaraldehyde (GA) solution 50 wt. % in H_2_O (CAS: 111-30-8), PBS, bovine serum albumin (BSA) (CAS: 9048-46-8), calcium chloride (CAS: 10043-52-4), sodium chloride (CAS: 7647-14-5), and Q (CAS: 6151-25-3) were purchased from Sigma-Aldrich, Taufkirchen, Germany. Tris-hydroxymethyl aminomethane (CAS: 77-86-1) was purchased from Serva, Heidelberg, Germany. GO powder was obtained by the Hummers’ modified method (~4.2 nm in thickness, ~10–20 layers) [72,95]. The primary Human Dermal Fibroblasts (HDF) cells were commercially acquired from Merck, Darmstadt, Germany (NACRES code: NA.81, UNSPSC code: 41106514, Biological source: Normal adult human skin, Growth mode: Adherent, Morphology: Fibroblast). As these are commercially obtained primary cells, no accession numbers are associated with a genetic database entry.

### 4.2. Preparation of PVA:Gel Hydrogels

For the fabrication of PVA:Gel hydrogels, two separate solutions were prepared. A PVA solution (7% *w*/*v*) was obtained by adding a known amount of PVA in deionized water and stirring at 90 °C until complete dissolution (~3 h). Similarly, a Gel solution (7% *w*/*v*) was obtained by dissolving gelatin powder in deionized water and stirring at 50 °C for 1 h, until a clear solution was formed. The two solutions were then mixed at different ratios: PVA:Gel 70:30, PVA:Gel 50:50, and PVA:Gel 30:70. A 0.1% *v*/*v* GA was added into the three mixtures, stirred until homogeneous solutions were obtained, transferred into Petri dishes, and subsequently frozen at −20 °C for 12 h. After the freezing process, to neutralize the GA, the hydrogels were immersed in 0.1 M glycine for 2 h. Finally, the obtained hydrogels were subjected to a lyophilization process (Labconco, Missouri, KC, USA).

### 4.3. Preparation of PVA:Gel:GO Hydrogels and Drug Loading

GO was synthesized using the modified Hummers’ method presented in our previous literature reports [72,95]. To obtain PVA:Gel:GO hydrogels, different concentrations of GO (0.1, 0.3, and 0.5% *w*/*v*) were dispersed into the PVA:Gel 30:70 solutions (prepared as mentioned above), under continuous stirring. To load the Q, a concentration of 0.12 mg/mL of Q was dispersed in each hydrogel blend and stirred until complete mixing of the solutions. The adsorption of hydrophobic Q onto the surface of GO nanoparticles was achieved via π–π stacking and hydrogen-bonding interactions. Additional hydrogen-bonding interactions can be achieved between Q and the PVA:Gel hydrogel network. The cross-linking process was realized with GA 0.1% *v*/*v*. A total of 6 formulations were obtained: PVA:Gel 0.1%GO, PVA:Gel 0.3%GO, PVA:Gel 0.5%GO, PVA:Gel 0.1%GO/Q, PVA:Gel 0.3%GO/Q, and PVA:Gel 0.5%GO/Q. The hydrogels were immersed in Petri dishes, frozen at −20 °C for 12 h, neutralized with 0.1 M glycine for 2 h, and lyophilized.

### 4.4. Characterization of the Hydrogels

The obtained hydrogels were characterized by Fourier transform infrared (FTIR) spectroscopy using a Nicolet iS50FT-IR spectrometer (Thermo Fisher Scientific, Waltham, MA, USA) equipped with an attenuated total reflectance (ATR) accessory using a diamond crystal. The measurements were performed in the 4000–500 cm^−1^ range at a resolution of 4 cm^−1^.

The surface morphology of the hydrogels was examined by scanning electron microscopy (SEM) using a Quanta Inspect F50 system (Eindhoven, The Netherlands) equipped with a field emission electron source (FEI Inspect F50, Eindhoven, The Netherlands), offering a resolution of 1.2 nm at 30 kV and 3 nm at 1 kV (BSE).

### 4.5. Determination of Water Content in Hydrogels

Initially, each hydrogel formulation was weighed. Next, the material samples were lyophilized and weighed again. The water content was determined as the difference between the hydrogel’s initial weight and its weight after lyophilization, using the following equation:

(1) $$Water\;content\left(\%\right)=\frac{W_{0}-W_{1}}{W_{0}}*100$$

where *W*_0_ and *W*_1_ represent the weight of the initial wet sample and the weight of the hydrogel at dry state, respectively.

### 4.6. Swelling Behavior

The swelling behavior of the hydrogels was evaluated from 5 to 240 min in SWF at 37 °C. The SWF contains 2% BSA, 0.02 M CaCl_2_, 0.4 M NaCl, and 0.08 M tris-hydroxymethyl aminomethane in deionized water (pH = 7.5). The hydrogels were cut into square shapes (1 cm × 1 cm) and immersed in SWF. At regular time intervals, the hydrogels were removed from the SWF, the excess water was removed using filter paper, and the samples were weighed immediately. The swelling ratio (%) was determined according to the following equation:

(2) $$Swelling\;ratio\;\left(\%\right)=\frac{W_{t}-W_{1}}{W_{1}}*100$$

where *W_t_* is the weight of the hydrogels at different time intervals, and *W*_1_ is the initial weight of the dry hydrogels.

### 4.7. In Vitro Drug Release Test

**Figure 14:**
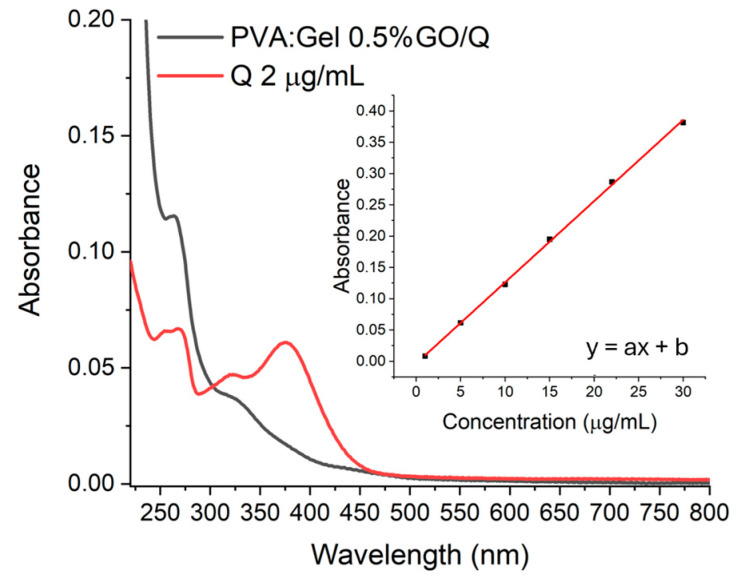
Absorption spectra of a 2 μg/mL solution of Q and Q released from the PVA:Gel 0.5%GO hydrogel after immersion in PBS for 5 min (without irradiation). The inset shows the calibration curve for Q and the equation of the calibration line (y = ax + b).

The in vitro release tests of Q from PVA:Gel:GO hydrogels were evaluated in PBS at pH 7.4 at room temperature. From the initial hydrogels, obtained in Section 4.3 in the Petri dish, 7 mm diameter hydrogel discs were cut. The height of the hydrogel was 5.12 ± 0.27 mm. For Q quantification, each hydrogel with a diameter of 7 mm was added to 1.5 mL of PBS. At specific time intervals (5, 10, 20, and 30 min), 1 mL from each flask was withdrawn, and the release profile of Q was determined using a Perkin Elmer Spectrophotometer (Boston, MA, USA, model Lambda 950) at 268 nm. Figure 14 shows the absorption spectrum of Q released by PVA:Gel 0.5%GO/Q after 5 min immersion in water and of a 2 μg/mL solution of Q. To quantify the concentration/mg of Q released from the hydrogel, a calibration curve (inset figure), using the equation embedded the inset figure, was obtained from the absorption spectra of six concentrations of Q, ranging from 1 to 30 μg/mL, measured in the same conditions as the release of Q from hydrogels.

**Figure 15:**
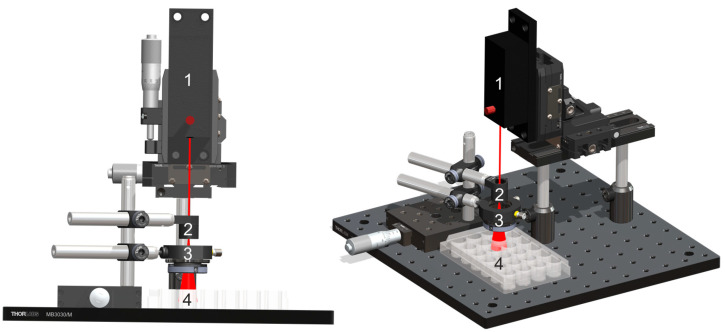
Experimental system for hydrogel irradiation to enhance the release of Q, created as a SolidWorks 2024 assembly. Legend: 1—laser diode; 2—beam expander; 3—spatial filter; 4—24-well plates.

To evaluate the photothermal Q release, the hydrogels with a diameter of 7 mm were immersed in 1.5 mL PBS and immediately exposed to laser radiation for 5, 10, 20, and 30 min. The NIR light source was a laser diode (Alphals GmbH, Göttingen, Germany; PicoPower LD-37550 model) emitting at 785 nm in continuous wave operation mode. The elliptical laser beam, initially measuring 1.6 × 1.8 mm, was expanded using a collimated lens (LDM-4015 model; manufacturer details not available) to dimensions of 9 × 7 mm. It was then spatially filtered with a diaphragm and reduced to a 7 mm diameter spot. The radiation was directed perpendicularly onto the hydrogels, resulting in a final power density of 29.8 mW/cm^2^. The experimental system for hydrogel irradiation is represented in Figure 15.

After each irradiation interval, the UV-Vis absorption spectrum of the released Q was recorded at 1 nm resolution between 200 and 400 nm with the Perkin Elmer Lambda 950 Spectrophotometer (PerkinElmer, Inc., Waltham, MA, USA). The optical path of the spectrophotometric cuvette used was 5 mm, and the total volume measured was 1 mL. The absorbance recorded at 268 nm was used together with the calibration curve to obtain the Q concentration released in PBS.

### 4.8. Assessment of Antimicrobial Activity

Experiments were carried out using five reference strains obtained from the American Type Culture Collection (ATCC, Manassas, VA, USA): Staphylococcus aureus ATCC 6538, Pseudomonas aeruginosa ATCC 15442, Escherichia coli ATCC 8739, and Candida albicans ATCC 10231. Antimicrobial susceptibility testing followed the CLSI guidelines [96]. Microbial suspensions were prepared at a concentration of 1.5 × 10^8^ CFU/mL from fresh cultures grown on solid medium for 15–18 h. These suspensions were standardized using the McFarland nephelometric method (0.5 for bacteria and 1.0 for fungi) and subsequently serially diluted to 10^5^. The inoculum volume was adjusted in proportion to the sample mass. Samples were exposed to microbial suspensions for 30 min, and then mixed thoroughly by vortexing, followed by five sequential tenfold dilutions to assess both the logarithmic and percentage reduction in viable microorganisms. Ten microliters of each dilution, in triplicate, were inoculated onto Mueller–Hinton agar for bacteria or Sabouraud agar for fungi. Plates were incubated at 36 ± 2 °C for 18–24 h, after which colonies were counted. All analyses were performed in triplicate.

The logarithmic reduction was calculated using the formula:

(3) Logarithmic reduction = Logarithmic reduction = lg A/B

where A = no. of viable organisms before treatment; B = no. of viable organisms after treatment.

The antimicrobial assay was designed to evaluate the immediate antimicrobial effect of the hydrogels upon contact with microorganisms. Long-term antimicrobial performance in simulated wound conditions will be investigated in future studies.

### 4.9. In Vitro Cytotoxicity Assessments

Cellular metabolic activity of the samples was evaluated using Human Dermal Fibroblasts (HDF). Prior to the assessment, HDF was maintained in fibroblast growth medium supplemented with 2 mM glutamine (Sigma-Aldrich, Taufkirchen, Germany), 10% heat-inactivated fetal bovine serum (FBS, Sigma-Aldrich, Taufkirchen, Germany), and 1% penicillin–streptomycin solution (50 µg/mL, Sigma-Aldrich, Taufkirchen, Germany). Cultures were incubated for 24 h at 37 °C under 5% CO_2_ and 95% relative humidity. Following this period, cells were rinsed with PBS, detached with trypsin (Sigma-Aldrich, Taufkirchen, Germany), and counted using Trypan Blue dye exclusion (Sigma-Aldrich, Taufkirchen, Germany) and a hemocytometer.

For both MTT and LDH assays, the optimal seeding density was 4 × 10^5^ cells. Cells were seeded at this density in 96-well culture plates and subsequently exposed to test samples or control conditions, followed by 24, 48, and 72 h of incubation under standard culture conditions. After each exposure time, the MTT assay was carried out by adding MTT reagent (Sigma, St. Louis, MO, USA) and incubating the cells for 4 h. The resulting formazan crystals were solubilized using the MTT solvent (Sigma, St. Louis, MO, USA) for 15 min at room temperature. Absorbance was then quantified at 570 nm with a Synergy™ HTX Multi-Mode Microplate Reader (Biotek, Winooski, VT, USA).

For cytotoxicity assessment, the LDH Cytotoxicity Detection Kit (Roche, Basel, Switzerland) was employed. Cells were treated with the LDH reagent mixture for 15 min at 37 °C, and enzymatic activity was measured spectrophotometrically at 492 nm with a 600 nm reference wavelength using the same microplate reader. PBS served as the negative control.

### 4.10. Total Association and Invasion of Bacteria on Fibroblasts

Using the HDF model, cells were cultured under the conditions previously described until reaching approximately 80% confluence. For the invasion assay, the culture medium of confluent HDF monolayers was replaced with pre-warmed fibroblast growth medium supplemented with 10% heat-inactivated FBS. A bacterial suspension of *S. aureus* ATCC 6538 (1.5 × 10^5^ CFU in 10 µL) was added to 990 µL of culture medium, followed by 50 µL of test samples. The plates were then incubated for 15 h at 37 °C, 5% CO_2_, and 95% relative humidity. PBS-treated cells served as the negative control.

Following incubation, the cells were washed three times with PBS. Half of the wells (in 24-well plates) were lysed with 100 µL of 0.1% Triton X for 15 min at 37 °C, and the lysates were plated on Mueller–Hinton agar to determine CFU counts. The remaining wells were first exposed to gentamicin sulfate (50 µg/mL) for 2 h at 37 °C and then washed six times with PBS before being lysed with 100 µL of 0.1% Triton X (15 min at 37 °C). The lysates were subsequently plated on Mueller–Hinton agar for CFU determination.

Statistical evaluations were performed using GraphPad Prism 9 (San Diego, CA, USA). Data were analyzed using the two-way ANOVA test with the Greenhouse–Geisser correction. The level of significance was set to *p* < 0.05.

## Figures and Tables

**Table 1:** Water content of hydrogels.

Hydrogel	Hydrated Hydrogel (g)	Dried Hydrogel (g)	Water Content (%)
PVA:Gel 70:30	5.73 ± 1.19	0.65 ± 0.12	88.66 ± 0.39
PVA:Gel 50:50	9.86 ± 0.78	0.62 ± 0.17	93.71 ± 1.44
PVA:Gel 30:70	19.21 ± 0.33	0.63 ± 0.06	96.72 ± 0.04
PVA:Gel:GO 0.1%	23.93 ± 1.03	2.12 ± 0.44	91.15 ± 1.53
PVA:Gel:GO 0.3%	21.67 ± 1.48	2.20 ± 0.24	89.86 ± 0.56
PVA:Gel:GO 0.5%	23.59 ± 0.61	2.19 ± 0.32	90.70 ± 1.39
PVA:Gel:GO 0.1%/Q	22.67 ± 0.91	1.75 ± 0.41	91.74 ± 1.84
PVA:Gel:GO 0.3%/Q	22.63 ± 0.75	1.67 ± 0.28	92.62 ± 0.89
PVA:Gel:GO 0.5%/Q	21.65 ± 0.55	1.80 ± 0.45	92.68 ± 1.48

**Table 2:** Therapeutic index of the scaffolds.

	PVA:Gel:GO	PVA:Gel:GO/Q
*S. aureus* ATCC 6538	0.67 ± 0.60	0.71 ± 0.58
*E. coli* ATCC 8739	2.21 ± 1.97	0.95 ± 0.78
*Ps. aeruginosa* ATCC 9027	1.74 ± 1.55	1.10 ± 0.91
*C. albicans* ATCC 10231	0.70 ± 0.62	0.79 ± 0.65

## Data Availability

The data presented in this study will be made available by the authors on request.
